# Microdomain calcium fluctuations as a colored noise process

**DOI:** 10.3389/fgene.2014.00376

**Published:** 2014-11-03

**Authors:** Frederic von Wegner, Nicolas Wieder, Rainer H. A. Fink

**Affiliations:** Medical Biophysics Group, Institute of Physiology and Pathophysiology, Heidelberg UniversityHeidelberg, Germany

**Keywords:** calcium microdomains, calcium signaling, molecular noise, stochastic simulation, Gillespie algorithm, chemical Langevin equation, Ornstein-Uhlenbeck process

## Abstract

Calcium ions play a key role in subcellular signaling as localized transients of the intracellular calcium concentration modify the activity of ion channels, enzymes and transcription factors, among others. The intracellular calcium concentration is inherently noisy, as diffusion, the transient binding to and dissociation from buffer molecules and stochastically gating calcium channels contribute to the fluctuations of the local copy number of Ca^2+^ ions. We study the properties of the fluctuating calcium concentration in sub-femtoliter volumes using an exact stochastic simulation algorithm and approximations to the exact stochastic solution. It is shown that the time course of the local calcium concentration represents a colored noise process whose autocorrelation time is a function of buffer kinetics and diffusion constants. Using the chemical Langevin description and the excess buffer approximation of the process, fast approximative algorithms and theoretical connections to the Ornstein-Uhlenbeck process are obtained. In a generic example, we show how calcium noise can couple to the dynamics of a single variable moving in a double-well potential, leading to a colored noise induced transition. Our work shows how a multitude of intracellular signaling pathways may be influenced by the inherent stochasticity of calcium signals, a key messenger in virtually any cell type, and how the calcium signal can be implemented efficiently in cellular signaling models.

## 1. Introduction

Calcium ions regulate intracellular signaling pathways in virtually all known cell types as many regulating proteins offer calcium binding sites by exhibiting negatively charged amino acid residues. Among these proteins, we can find calcium-regulated ion channels, enzymes and transcription factors, some of them clustered in families such as the EF-hand proteins (Bhattacharya et al., 2004). Due to the strong compartmentalization of the intracellular space, the local calcium concentration can differ significantly between two spots within the same cell. The most obvious example may be that of a small intracellular domain next to a calcium channel located within the plasma membrane or the endoplasmic reticulum membrane. Given the strong extra-/intracellular concentration gradient for calcium, channel opening leads to a quick rise of the local calcium concentration. The spatiotemporal extent of this calcium increase is determined by the kinetic constants and the diffusibility of the buffers involved (Smith et al., 1998; Jiang et al., 1999; Uttenweiler et al., 2002). Moreover, the non-linear interplay of different calcium release und re-uptake mechanisms generate more complex global calcium patterns such as traveling waves and oscillations. All of the aforementioned phenomena can be modeled deterministically using ordinary and partial differential equations. However, closer inspection of the conditions encountered in calcium microdomains shows that the subcellular calcium concentration must contain a certain amount of stochasticity as induced by the stochastic nature of diffusion, transient chemical binding and the gating of calcium channel proteins. The magnitude of the fluctuations, and therefore their possible physiological relevance, depends on the reaction volumes considered. Ignoring contributions from calcium channels and given an intracellular calcium concentration of approximately 100 nM in a resting cell, we expect to find approximately 60 Ca^2+^ ions in a volume of 10^−15^ liter (1 fl). Fluctuations around the mean value will have a *SD* given by the square root of the mean value, i.e., the number of Ca^2+^ ions will be approximately $60\pm \sqrt{60}$ ions. Femtoliter volumes are of special interest for several reasons. First, localized calcium transients such as calcium sparks, puffs and quarks occupy femtoliter volumes, as well as their functional targets such as ion channel clusters and mitochondria (Cheng and Lederer, 2008). Second, current measurement devices for calcium fluorescence microsopy, such as confocal and multiphoton laser microscopes, collect fluorescence from volumes of approximately 1 fl. Therefore, computational models of reactions occurring within these volumes yield important data that can help to design new experiments and to interpret experimental results.

Until recently, partial differential equations were the main tool to build reaction-diffusion models of calcium microdomains (Smith et al., 1998; Jiang et al., 1999; Uttenweiler et al., 2002). The PDE approach is completely deterministic and ignores deviations from equilibrium concentrations and deterministic solutions. In a previous paper, we have shown computationally that fluctuations of calcium and other reactants lead to highly variable responses of calcium-sensitive signaling pathways (von Wegner and Fink, 2010), suggesting an important role of the stochastic aspects of calcium dynamics. Other authors have studied calcium signaling on a whole cell level using approximations of the exact stochastic model (Zhang et al., 2004; Li et al., 2005; Manninen et al., 2006; Zhu et al., 2007; Choi et al., 2010). The use of different stochastic models in the context of microdomain calcium signaling has been summarized in recent review articles (Wieder et al., 2011; von Wegner et al., 2012). In the present paper, we study the properties of the fluctuating calcium concentration in sub-femtoliter volumes using Gillespie's exact stochastic simulation algorithm as well as stochastic approximations. It is shown that the time course of the local calcium concentration represents a colored noise process whose noise color, i.e., its autocorrelation time is a function of the kinetic constants of the buffer species considered. Using the chemical Langevin equation, theoretical connections to the Ornstein-Uhlenbeck process are obtained. A possible role of calcium noise color in the gating behavior of a calcium-sensitive ion channel is illustrated using a colored noise driven bistable dynamical system as a generic representation of a calcium noise-driven ion channel. Our results show how a multitude of intracellular signaling pathways may be influenced by the inherent stochasticity of calcium signals, a key messenger in virtually any cell type, and how the stochastic calcium signal can be implemented efficiently in computational models of cellular calcium signaling.

## 2. Deterministic and stochastic description of calcium dynamics

In the following, we give a mathematical description of deterministic and stochastic calcium dynamics. We will denote the time-dependent concentrations of calcium ions, free buffer molecules and calcium-buffer complexes as [Ca^2+^]_*t*_, [B]_*t*_, and [CaB]_*t*_, respectively. For the sake of readability, we substitute these bracketed expressions by *x*_*t*_, *y*_*t*_, and *z*_*t*_ in formulas. When a stochastic framework is used, we will work with molecule counts rather than concentrations and denote these as capital letters, e.g., the number of free calcium ions in a given volume *V* is denoted as *X*_*t*_ = *x*_*t*_ × *V* (analogously for *Y*_*t*_ and *Z*_*t*_). Equilibrium concentrations (or molecule counts) are denoted by a tilde, e.g., the free calcium concentration at chemical equilibrium is written as $\tilde{x}$, and the corresponding number of calcium ions at equilibrium is denoted $\tilde{X}$. When a system contains *M* different buffer species B_1_, …, B_*M*_, we write their time-dependent concentrations with parenthesized superscripts *y*^(1)^_*t*_, …, *y*^(*M*)^_*t*_, and their molecule counts as *Y*^(1)^_*t*_, …, *Y*^(*M*)^_*t*_. Initial concentrations receive a 0-subscript, e.g., *x*_0_ is the initial concentration of free calcium ions at *t* = 0. The total calcium concentration is [Ca]_*T*_ = *x*_*T*_ = [Ca^2+^] + [CaB], and the total buffer concentration is B_*T*_ = *y*_*T*_ = [B] + [CaB].

### 2.1. Deterministic dynamics

Consider a simple reaction system containing calcium ions, Ca^2+^, and a single calcium buffer *B*. In real biological systems and in experimental settings, the buffer often is a protein, calmodulin for instance, or a small organic molecule, e.g., EGTA. The following reaction scheme describes the system :

(1) $${\text{Ca}}^{2+}+\text{B}\overset{k^{+}}{\underset{k^{-}}{⇌}}\text{CaB}$$

with reaction velocities *v*^+^_*t*_ = *k*^+^[Ca^2+^]_*t*_[B]_*t*_ and *v*^−^ = *k*^−^[CaB]_*t*_, defined via the reaction rate constants *k*^+^, *k*^−^.

The equilibrium condition $k^{+}[\tilde{{\text{Ca}}^{2+}}][\tilde{\text{B}}]=k^{-}[\tilde{\text{CaB}}]$ leads to:

(2) $$[\tilde{\text{CaB}}]=\frac{{\left[\text{B}\right]}_{T}}{1+\frac{K_{D}}{\left[\tilde{{\text{Ca}}^{2+}}\right]}}.$$

Thus, all equilibrium concentrations depend solely on the total calcium and buffer concentrations *x*_*T*_ and *y*_*T*_, the buffer dissociation constant $K_{D}=\frac{k^{-}}{k^{+}}$, and the nominal free calcium concentration at equilibrium ($[\tilde{{\text{Ca}}^{2+}}]=\tilde{x}$).

The following ordinary differential equation describes the deterministic kinetics of the free calcium concentration:

(3) $${\dot{x}}_{t}=-k^{+}x_{t}y_{t}+k^{-}z_{t}.$$

Given fixed total concentrations [Ca^2+^]_*T*_ and [B]_*T*_, the single buffer system has only one independent variable, and the dynamics of the other two variables is given by *ẏ*_*t*_ = *ẋ*_*t*_ and *ż*_*t*_ = −*ẋ*_*t*_. Using the definitions of *y*_*T*_ and *x*_*T*_, we can rewrite the dynamics as the non-linear ODE:

$$\begin{array}{l} {\dot{x}}_{t}=-k^{+}x_{t}[y_{T}-x_{T}+x_{t}]+k^{-}[x_{T}-x_{t}]\\ \;=-k^{+}\left(x_{t}[x_{t}+y_{T}-x_{T}+K_{D}]-K_{D}x_{T}\right). \end{array}$$

We will apply the excess buffer approximation to this system to obtain a linear ODE, and in subsequent sections derive considerably simplified stochastic versions of the kinetic equations.

In the present work, we have used the physico-chemical properties of Ca^2+^ ions and buffers as published elsewhere and as given in Table 1, along with references. We provide the variable simulation parameters such as reaction volumes in the corresponding results sections.

**Table 1:** **Physiological and artificial calcium buffers used in simulations**.

**Buffer**	**B_*T*_ [μM]**	**k^+^ [μM^−1^ ms^−1^]**	**k^−^ [ms^−1^]**	**K_*D*_ [μM]**	$\alpha =\frac{K_{D}}{B_{T}}$	$CV=\frac{\sigma }{\mu }$	**References**
CaM	24	0.1	0.038	0.380	0.016	0.001	Smith et al., 1998
TnC	140	0.12	0.023	0.192	0.001	<0.000	Uttenweiler et al., 2002
PV	1000	0.25	0.001	0.004	<0.000	<0.000	Jiang et al., 1999
EGTA	100	0.0015	0.00094	0.627	0.006	<0.000	Uttenweiler et al., 2002
Fluo-4	100	0.236	0.175	0.742	0.007	<0.000	Uttenweiler et al., 2002
OGB-5N	100	0.17	5.6	32.941	0.329	<0.000	Novo et al., 2003

*B_T_, total buffer concentration; k^+^, k^−^, kinetic rate constants; K_D_, dissociation constant; α, EBA approximation; CV, coefficient of variation (from Gillespie's algorithm, T_max_ = 100 ms, V = 1 fl); CaM, calmodulin; TnC, troponin C; PV, parvalbumin; EGTA, ethylene glycol tetraacetic acid; OGB-5N, Oregon Green BAPTA-5N*.

### 2.2. The excess buffer approximation (EBA)

In biological systems, at rest, the free calcium concentration is approximately 100 nM, whereas the total buffer concentration lies in the micromolar range (Smith et al., 1998; Jiang et al., 1999; Uttenweiler et al., 2002; Novo et al., 2003; von Wegner and Fink, 2010). The free buffer concentration can therefore often be assumed to be constant, i.e., *y*_*t*_ ≈ ỹ. In biological systems, the total buffer concentration is usually large enough to assume a constant free buffer concentration [B], i.e., only a small fraction of the total buffer molecules is bound to Ca^2+^. Thus, under the EBA assumption (Heinemann et al., 1986; Smith et al., 2001; Fall et al., 2002), the free buffer concentration [B] is assumed to be identical to the equilibrium concentration, i.e., *y*_*t*_ = ỹ. In the original derivation of the EBA, calcium and buffer diffusion were considered close to a calcium channel pore. The approximation was considered valid for small ratios *K*_*D*_/[B]_*T*_, i.e., when the total buffer concentration is [B]_*T*_ large, or when dealing with high-affinity buffers. In the current work, additional calcium influx through a calcium channel is not considered, and therefore, all systems are far from buffer saturation. Using the approximation *y*_*T*_ = *ỹ* + *z*_*t*_, valid under the EBA assumption, the simplified deterministic dynamics can be rewritten as:

(4) $$\begin{array}{l} {\dot{x}}_{t}=-k^{+}x_{t}\tilde{y}+k^{-}[x_{T}-x_{t}]\\ \;=-(k^{+}\tilde{y}+k^{-})x_{t}+k^{-}x_{T}. \end{array}$$

The free calcium concentration [Ca^2+^] follows an inhomogeneous ODE with constant coefficients

(5) $$\begin{array}{l} K_{1}=k^{+}\tilde{y}+k^{-}\\ K_{2}=k^{-}x_{T} \end{array}$$

and with initial condition [Ca^2+^]_0_ = *x*_0_:

(6) $${\dot{x}}_{t}=-K_{1}x_{t}+K_{2}.$$

The equilibrium calcium concentration $\tilde{x}$ in terms of the new ODE coefficients is $\tilde{x}=\frac{K_{2}}{K_{1}}$. The straightforward solution to Equation 6 is

(7) $$x_{t}=(x_{0}-\tilde{x})\exp(-K_{1}t)+\tilde{x}.$$

Clearly, the EBA-simplified dynamics predicts a mono-exponential relaxation of the single buffer system to calcium elevations that do not violate the EBA-assumption, in other words, the Ca^2+^ peak must not alterate the free buffer concentration significantly.

### 2.3. Stochastic dynamics

In this section, we introduce stochastic descriptions of the simplified calcium dynamics. We first review Gillespie's original algorithm for exact stochastic simulations without re-deriving all details. Extended presentations of the algorithm for general systems and for calcium dynamics in particular can be found in the literature (Gillespie, 1977, 2007; von Wegner and Fink, 2010; Wieder et al., 2011; von Wegner et al., 2012). Subsequently, we formulate the chemical Langevin equation for the model systems at hand, i.e., a single buffer system, a single buffer system with calcium diffusion, and a multi-buffer system without diffusion. Last, we introduce the Ornstein-Uhlenbeck process as the generic Gaussian, exponentially correlated colored noise process.

#### 2.3.1. Exact stochastic simulation algorithm—Gillespie's algorithm (SSA)

Systems of chemical reactions can be represented by multivariate Markov processes (Gardiner, 2009). In the case of chemical reaction systems, each variable describes the time-dependent copy number of exactly one molecular species. The Gillespie algorithm generates exact sample paths of the Markov process alternating between two sampling steps. First, a sample of the state-dependent waiting time distribution until the next reaction event is generated. In a second step, exactly one reaction is selected from the list of all possible reactions according to their relative reaction propensities (Gillespie, 2000, 2007). We will restrict the description of the algorithm to the notions that will be used in the following sections. In order to correctly capture reaction probabilities in small volumes, the deterministic reaction rate constants *k*^+^, *k*^−^ have to be transformed into the corresponding stochastic rate constants *c*^+^, *c*^−^. For mono- and bimolecular reactions, the transformations are elementary and only involve a scaling by the reaction volume *V* for the bimolecular reaction: $c^{+}=\frac{k^{+}}{V}$, *c*^−^ = *k*^−^, using the notation used in Equation 1. As we will exclusively deal with systems of mono- and bimolecular reactions, all non-vanishing stoichiometric coefficients *v*_*ji*_ are either −1 or +1. The coefficient *v*_*ji*_ reflects the change in the copy number of reactant *i* due to the reaction indexed by *j*. Diffusion is implemented as derived in Elf et al. (2003) and as implemented for calcium microdomains in von Wegner and Fink (2010). As the waiting time to the next reaction event is a random variable, Gillespie's algorithm generates non-equidistant sample paths that are linearly interpolated prior to further processing. We chose an interpolation interval of *dt* = 0.01 ms that is identical to the integration time step *dt* used for the chemical Langevin equation and the Ornstein-Uhlenbeck process.

#### 2.3.2. The chemical Langevin equation (CLE)

The CLE approach combines the deterministic dynamics as described by reaction rates with the stochastic description of the Gillespie algorithm. Mathematically, it is based on the approximation of a Poisson-distributed random variable, representing the number of reaction events in a short interval of time, by a normally distributed random variable, thus achieving the connection to the general Langevin equation (Gillespie, 2000). The dynamics are driven by a stochastic process *dW*_*t*_. The terms *dW*_*t*_ represent the stochastic increments of a Brownian motion (also called a standard Wiener process) (Gardiner, 2009). The increments are normally distributed as *W*_*t+dt*_ − *W*_*t*_ ~ 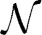
(0, *dt*) and are pairwise uncorrelated. Furthermore, the initial condition is *W*_0_ = 0. Algorithmically, such increments are computed using normally distributed pseudo-random numbers with mean μ = 0 and variance σ^2^ = *dt*. Equivalently, pseudo-random numbers drawn from a standard normal distribution (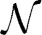
(0, 1)) can be multiplied by the factor $\sqrt{dt}$ to obtain the same process. Explicit examples for systems as discussed here are given in von Wegner et al. (2012); Higham (2001).

For the *single buffer system*, Equation 1, the chemical Langevin equation reads:

(8) $$\begin{array}{l} dX_{t}=-c^{+}\;X_{t}\;Y_{t}\;dt+c^{-}\;Z_{t}\;dt-\sqrt{c^{+}\;X_{t}\;Y_{t}}\;dW_{t}^{\left(1\right)}\\ \;+\;\sqrt{c^{-}\;Z_{t}}\;dW_{t}^{\left(2\right)} \end{array}$$

where *dW*^(1)^_*t*_, *dW*^(2)^_*t*_ are mutually independent, standard Wiener processes.

Second, consider a *single buffer system with calcium diffusion*, i.e., a system where calcium ions can diffuse into and out of the simulation volume. We will use a “constant pool” assumption, meaning that the simulation voxel is surrounded by a volume containing all molecular species at equilibrium concentrations. Thus, Ca^2+^ ions diffusing into and out of the simulation voxel change the simulations voxel's calcium concentration only, leaving the influx rate of calcium ions constant over time while the efflux rate varies. As diffusion is a monomolecular reaction, the deterministic and stochastic rate constants *k*_*d*_ and *c*_*d*_ are identical and given by $k_{d}=c_{d}=\frac{D}{L^{2}}$ (Elf et al., 2003; von Wegner and Fink, 2010). Here, *D* is the diffusion constant of calcium ions and the voxel volume is *V* = *L*^3^.

(9) $$\begin{array}{l} {\text{Ca}}^{2+}+\text{B}\overset{k^{+}}{\underset{k^{-}}{⇌}}\text{CaB}\\ \;{\text{Ca}}^{2+}\overset{k_{d}}{\underset{k_{d}}{⇌}}\emptyset \end{array}$$

The chemical Langevin equation for this system is:

(10) $$\begin{array}{l} dX_{t}=-c^{+}\;X_{t}\;Y_{t}\;dt+c^{-}\;Z_{t}\;dt-c_{d}\;X_{t}\;dt+c_{d}\;\tilde{X}\;dt\\ \;-\;\sqrt{c^{+}\;X_{t}\;Y_{t}}\;dW_{t}^{\left(1\right)}+\sqrt{c^{-}\;Z_{t}}\;dW_{t}^{\left(2\right)}-\sqrt{c_{d}\;X_{t}}\;dW_{t}^{\left(3\right)}\\ \;+\;\sqrt{c_{d}\;\tilde{X}}\;dW_{t}^{\left(4\right)}. \end{array}$$

Finally, we consider a *multiple buffer system* containing calcium ions and *M* different calcium buffers B_*j*_, *j* = 1, …, *M*:

(11) $$\begin{array}{l} \;{\text{Ca}}^{2+}+{\text{B}}_{1}\overset{k_{1}^{+}}{\underset{k_{1}^{-}}{⇌}}{\text{CaB}}_{1}\\ \;\vdots \\ {\text{Ca}}^{2+}+{\text{B}}_{M}\overset{k_{M}^{+}}{\underset{k_{M}^{-}}{⇌}}{\text{CaB}}_{M} \end{array}$$

The corresponding chemical Langevin equation reads:

(12) $$\begin{array}{l} dX_{t}=-\sum_{j\;=\;1}^{M}c_{j}^{+}\;X_{t}\;Y_{t}^{\left(j\right)}\;dt+\sum_{j\;=\;1}^{M}c_{j}^{-}\;Z_{t}^{\left(j\right)}\;dt\\ \;-\;\sum_{j\;=\;1}^{M}\sqrt{c_{j}^{+}\;X_{t}\;Y_{t}^{\left(j\right)}}\;dW_{t}^{\left(j,1\right)}\text{​}+\sum_{j\;=\;1}^{M}\sqrt{c_{j}^{-}\;Z_{t}^{\left(j\right)}}\;dW_{t}^{\left(j,2\right)} \end{array}$$

where any combination *dW*^(*j*,1)^_*t*_, *dW*^(*j*,2)^_*t*_ is a pair of mutually independent Wiener processes.

#### 2.3.3. The Ornstein-Uhlenbeck process (OUP)

The Ornstein-Uhlenbeck process uses a minimum of parameters to yield a stochastic process (*X*_*t*_)_*t*≥0_, *X*_*t*_ ∈ ℝ, *t* ∈ ℝ_≥0_, which is stationary, Gaussian and exponentially autocorrelated in time. In one dimension, it is fully described by an initial condition *X*_0_ and three parameters, the mean μ, the autocorrelation time τ, and the volatility σ. In the context of general stochastic processes, the term volatility is used to indicate a time-dependent parameter σ_*t*_, leading to time-varying values of the variance of the process. Whereas σ is constant only for the more simple examples of stochastic processes, e.g., Brownian motion or the Ornstein-Uhlenbeck process, in general σ_*t*_ is itself a stochastic process. To take this feature into account from the beginning, we will use the term noise volatility. The variance of the Ornstein-Uhlenbeck process is constant and is given by $Var(X_{t})=\frac{\sigma^{2}\tau }{2}$ (Gardiner, 2009). Both, buffered calcium dynamics and the OU-process are mean-reverting processes, i.e., deviations from the mean value μ drive the system back toward the mean. The autocorrelation time τ, or “noise color,” of the process quantifies how fast the system responds to deviations from the equilibrium value. Large τ values indicate strongly colored noise, i.e., a stochastic process with a slowly decaying exponential autocorrelation function. Small values of τ reflect short-lived fluctuations and the resulting noise approximates Gaussian white noise with τ approaching zero (Gillespie, 1996). The following stochastic differential equation describes the Ornstein-Uhlenbeck process:

(13) $$dX_{t}=-\frac{1}{\tau }(X_{t}-\mu )\;dt+\sigma \;dW_{t}.$$

In the following, we will try to recast the chemical Langevin equations presented above into forms similar to the Ornstein-Uhlenbeck process, where possible.

All simulations in the manuscript were coded and run in python 2.7.6 and can be obtained from the corresponding author by mail request. For larger simulation runs, we recommend the use of compiled code, e.g., Cython or C/C++.

## 3. Results

### 3.1. Approximate stochastic kinetics

In this section, the chemical Langevin equation is formulated for different systems, in particular for (i) single buffer systems without diffusion, (ii) single-buffer systems with calcium diffusion, and (iii) systems with several buffers but no diffusion. Application of the excess buffer approximation, *Y*_*t*_ = *Ỹ* leads to approximations of the Langevin equation. These approximations have a functional form similar to the Ornstein-Uhlenbeck process, however, the process parameters contain non-stationary terms. Substituting the equilibrium values of relevant molecular concentrations, non-stationarities can be eliminated and still, good approximations to exact simulation results are obtained. Furthermore, approximations of the CLE lead to estimates of the autocorrelation time τ and for the volatility σ of the process.

#### 3.1.1. A single buffer system

For the single buffer system described by Equation 1, we substitute the EBA assumption *Y*_*t*_ = *Ỹ* and *Z*_*t*_ = *X*_*T*_ − *X*_*t*_ in Equation 8. Next, we make use of the fact that the sum of two independent, Gaussian random variables with means μ_1_, μ_2_ and variances σ^2^_1_, σ^2^_2_ yields another Gaussian random variable: 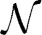
(μ_1_, σ^2^_1_) + 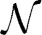
(μ_2_, σ^2^_2_) ~ 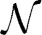
(μ_1_ + μ_2_, σ^2^_1_ + σ^2^_2_) (Gillespie, 2000). Applying this formula to the mutually uncorrelated Brownian motion terms *dW*^(*j*)^_*t*_ of the chemical Langevin equation, we obtain a single Brownian motion. The simplified Langevin equation of the single buffer system is then given by:

$$dX_{t}=-(c^{+}\;\tilde{Y}+c^{-})\;X_{t}\;dt+c^{-}\;X_{T}\;dt+\sqrt{c^{+}\;X_{t}\;\tilde{Y}+c^{-}\;Z_{t}}\;dW_{t}.$$

Using the definitions of the constants *K*_1_ and *K*_2_ (Equation 5), the analogous expressions in the stochastic framework are given by *C*_1_ = *c*^+^*Ỹ* + *c*^−^ and *C*_2_ = *c*^−^*X*_*T*_. We obtain:

(14) $$dX_{t}=-C_{1}\;(X_{t}-\frac{C_{2}}{C_{1}})\;dt+\sqrt{c^{+}\;X_{t}\;\tilde{Y}+c^{-}\;Z_{t}}\;dW_{t}.$$

Now, using the fact that the equilibrium calcium concentration [Ca^2+^]_*eq*_ is related to $\tilde{X}$ by ${\left[{\text{Ca}}^{2+}\right]}_{eq}\;V=\tilde{X}=\frac{C_{2}}{C_{1}}$ and defining $\tau =\frac{1}{C_{1}}$ and the time-dependent volatility $\sigma_{t}=\sqrt{c^{+}\;X_{t}\;\tilde{Y}+c^{-}\;Z_{t}}$, we get:

(15) $$dX_{t}=-\frac{1}{\tau }\;(X_{t}-\tilde{X})\;dt+\sigma_{t}\;dW_{t}.$$

The last expression is almost identical to an Ornstein-Uhlenbeck process, Equation 13, except from the non-stationary term σ_*t*_.

#### 3.1.2. Single buffer with calcium diffusion

Consider a simple reaction system containing calcium ions, Ca^2+^, and a calcium buffer B. For the single buffer system with calcium diffusion, the CLE is transformed as:

(16) $$\begin{array}{l} dX_{t}=-c^{+}\;X_{t}\;\tilde{Y}\;dt+c^{-}\;(X_{T}-X_{t})\;dt-c_{d}\;(X_{t}-\tilde{X})\;dt\\ \;-\;\sqrt{c^{+}\;X_{t}\;\tilde{Y}}\;dW_{t}^{\left(1\right)}+\sqrt{c^{-}\;Z_{t}}\;dW_{t}^{\left(2\right)}-\sqrt{c_{d}\;X_{t}}\;dW_{t}^{\left(3\right)}\\ \;+\;\sqrt{c_{d}\;\tilde{X}}\;dW_{t}^{\left(4\right)}\\ \;=\;(-C_{1}\;X_{t}+C_{2})\;dt-c_{d}\;(X_{t}-\tilde{X})\;dt\\ \;+\;\sqrt{c^{+}\;X_{t}\;\tilde{Y}+c^{-}\;Z_{t}+c_{d}\;X_{t}+c_{d}\;\tilde{X}}\;dW_{t} \end{array}$$

The term −*c*_*d*_ (*X*_*t*_ − $\tilde{X}$) represents diffusion into and out of the simulation volume under the constant pool assumption. The term −*c*_*d*_ *X*_*t*_ is a function of the non-stationary calcium concentration *X*_*t*_ and represents the diffusive flux out of the simulation volume. Assuming an invariant equilibrium calcium concentration (a constant pool) outside of the simulation volume, the diffusive flux into the simulation volume, *c*_*d*_ $\tilde{X}$, is constant. Using *C*_1,2_ as defined above, we get:

(17) $$\begin{array}{l} dX_{t}=-(C_{1}+c_{d})(X_{t}-\tilde{X})\;dt\\ \;+\;\sqrt{c^{+}\;X_{t}\;\tilde{Y}+c^{-}\;Z_{t}+c_{d}\;(X_{t}+\tilde{X})}\;dW_{t}. \end{array}$$

#### 3.1.3. Multiple buffer systems

Consider a reaction system containing calcium ions and *M* different calcium buffers B_*j*_, *j* = 1, …, *M*. For multiple buffer systems, the following substitutions are applied to the chemical Langevin equation (Equation 12):

(18) $$\begin{array}{l} Y_{t}^{\left(j\right)}={\tilde{Y}}^{\left(j\right)}\\ Z_{t}^{\left(j\right)}=X_{T}-X_{t}-\sum_{\begin{array}{c} i\;=\;1\\ i\;\neq \;j \end{array}}^{M}Z_{t}^{\left(i\right)}. \end{array}$$

This last substitution, Equation 18, may seem to be of little help as we substitute the single, time-dependent variable *Z*^(*j*)^_*t*_ by a more complicated expression involving the time-dependent variables *X*_*t*_ as well as all other CaB concentrations *Z*^(*i*)^_*t*_, *i* ≠ *j*; however, this representation leads to *C*_1,2_ terms that can easily be interpreted as modifications to the single buffer system. First, the corresponding CLE in the excess buffer approximation regime reads:

(19) $$\begin{array}{l} dX_{t}=-\left(\sum_{j\;=\;1}^{M}c_{j}^{+}{\tilde{Y}}^{\left(j\right)}\right)X_{t}dt+\sum_{j\;=\;1}^{M}c_{j}^{-}\left(X_{T}-X_{t}-\sum_{\begin{array}{c} i\;=\;1\\ i\;\neq \;j \end{array}}^{M}Z_{t}^{\left(i\right)}\right)dt\\ \;+\sqrt{\sum_{j\;=\;1}^{M}c_{j}^{+}X_{t}{\tilde{Y}}^{\left(j\right)}+c_{j}^{-}Z_{t}^{\left(j\right)}}dW_{t}^{\left(j\right)}. \end{array}$$

To simplify, we will introduce a more compact notation. We will reuse the variable names *C*_1,2_—however, it is important to note that in the context of multiple buffers, *C*_2_ is not a constant term. This represents another source of non-stationarity. Defining

$$\begin{array}{l} C_{1}=\sum_{j\;=\;1}^{M}c_{j}^{+}{\tilde{Y}}^{\left(j\right)}+c_{j}^{-}\\ C_{2}=\sum_{j\;=\;1}^{M}c_{j}^{-}\left(X_{T}-\sum_{\begin{array}{c} i\;=\;1\\ i\;\neq \;j \end{array}}^{M}Z_{t}^{\left(i\right)}\right) \end{array}$$

as well as $\tau =\frac{1}{C_{1}}$ and $\mu_{t}=\frac{C_{2}}{C_{1}}$, we can rewrite:

(20) $$dX_{t}=-\frac{1}{\tau }(X_{t}-\mu_{t})dt+\sqrt{\sum_{j\;=\;1}^{M}c_{j}^{+}X_{t}{\tilde{Y}}^{\left(j\right)}+c_{j}^{-}Z_{t}^{\left(j\right)}}dW_{t}.$$

Again, a functional form similar to an Ornstein-Uhlenbeck process, but with remaining non-stationary terms is obtained.

#### 3.1.4. Estimated autocorrelation times τ and noise volatilities σ

In order to approximate the solutions of Gillespie's exact simulation algorithm and the chemical Langevin equation by a simple Ornstein-Uhlenbeck process, the non-stationarity contained in the terms μ_*t*_ and σ_*t*_ has to be eliminated by approximation. We observe that non-stationarity is introduced by the time-dependent reactant concentrations, *X*_*t*_ and *Z*_*t*_. In the following, we substitute these expressions with their equilibrium values $\tilde{X}$ and $\tilde{Z}$ to obtain time-stationary expressions.

In the case of a *single buffer without diffusion*, the mean value is $\mu =\frac{C_{2}}{C_{1}}$, and the estimated autocorrelation time in the excess buffer approximation is given by:

(21) $$\begin{array}{l} \tau ={\left(c^{+}\tilde{Y}+c^{-}\right)}^{-1}\\ \;=\frac{1}{C_{1}}. \end{array}$$

In terms of the deterministic rate constants, the total buffer concentration [B]_*T*_ and the equilibrium calcium concentration $\left[\tilde{{\text{Ca}}^{2+}}\right]$, a more explicit expression for τ is:

(22) $$\tau =\frac{1}{k^{+}{\left[\text{B}\right]}_{T}\left(1-{\left(1+\frac{K_{D}}{\left[\tilde{{\text{Ca}}^{2+}}\right]}\right)}^{-1}\right)+k^{-}}.$$

Using the same approach to eliminate the non-stationary expression σ_*t*_ in Equation 15 using equilibrium concentrations, we obtain the constant term:

(23) $$\sigma =\sqrt{c^{+}\tilde{X}\tilde{Y}+c^{-}\tilde{Z}}.$$

The resulting variance of the calcium noise in the Ornstein-Uhlenbeck representation is then given by $Var(X)=\sigma^{2}\frac{\tau }{2}$.

In the case of a *single buffer with calcium diffusion*, we obtained stationary expressions for $\mu =\frac{C_{2}}{C_{1}}$ and $\tau =\frac{1}{C_{1}}$, the mean calcium concentration and the autocorrelation time, respectively. When substituting equilibrium reactant concentrations in the non-stationary expression for σ_*t*_, Equation 17, we get the stationary value σ for the approximating Ornstein-Uhlenbeck process:

$$\sigma =\sqrt{\left(c^{+}\;\tilde{Y}+2c_{d}\right)\;\tilde{X}+c^{-}\;\tilde{Z}}.$$

In the case of *multiple buffers without diffusion*, both parameters, μ_*t*_ and σ_*t*_ contain non-stationary terms as shown in Equation 20. Applying the equilibrium approximation, we get:

$$\mu =\frac{\sum_{j\;=\;1}^{M}c_{j}^{-}\left(X_{T}-\sum_{\begin{array}{c} i\;=\;1\\ i\;\neq \;j \end{array}}^{M}{\tilde{Z}}^{\left(i\right)}\right)}{\sum_{j\;=\;1}^{M}c_{j}^{+}{\tilde{Y}}^{\left(j\right)}+c_{j}^{-}}.$$

and

$$\sigma =\sqrt{\sum_{j\;=\;1}^{M}c_{j}^{+}\;\tilde{X}\;{\tilde{Y}}^{\left(j\right)}+c_{j}^{-}\;{\tilde{Z}}^{\left(j\right)}}.$$

Finally, the estimated autocorrelation time is

$$\tau =\frac{1}{\sum_{j\;=\;1}^{M}c_{j}^{+}{\tilde{Y}}^{\left(j\right)}+c_{j}^{-}}.$$

**Figure 1:**
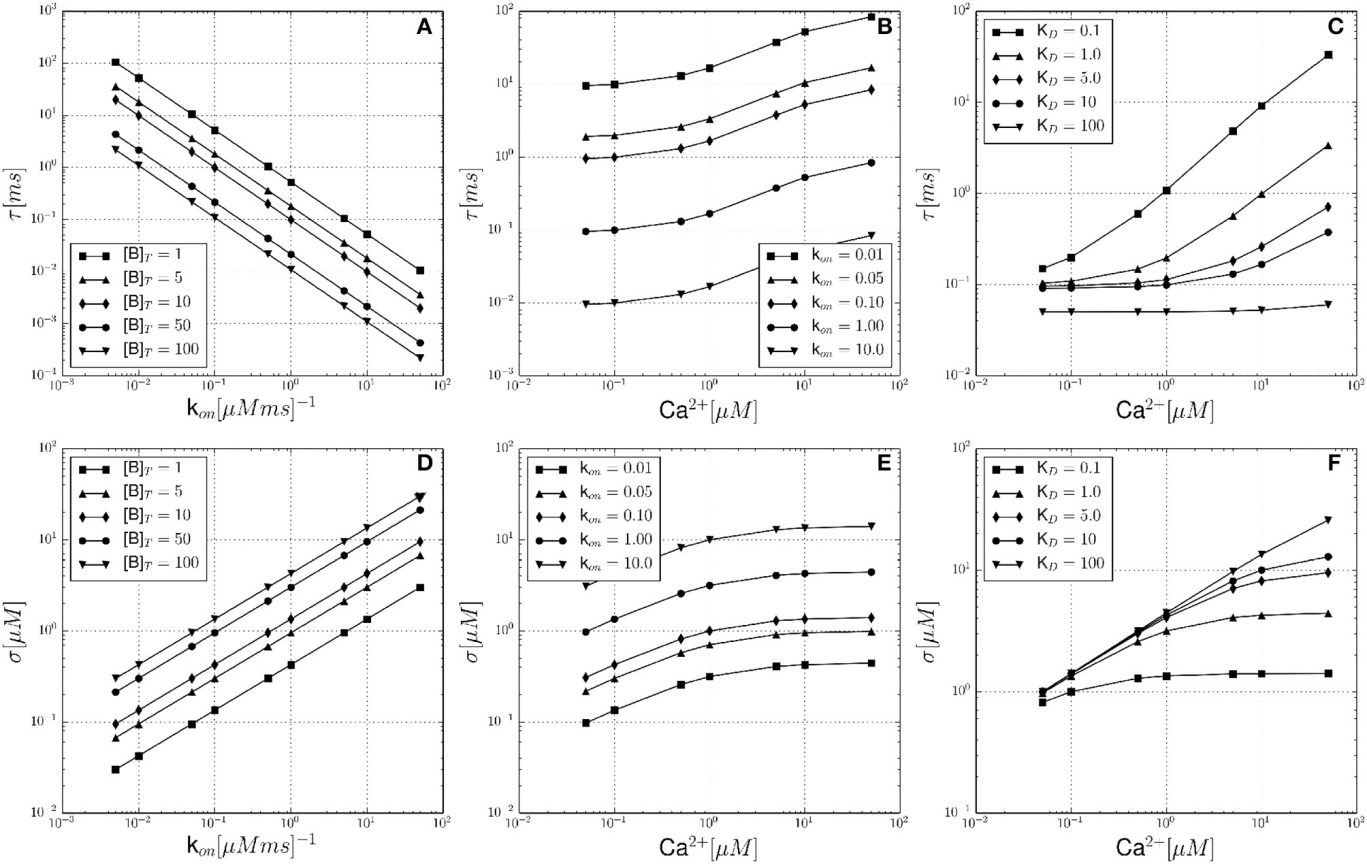
**Theoretical noise autocorrelation time τ and noise volatility σ for the single buffer system without diffusion. (A)** Shows the dependency of the noise autocorrelation time τ on the calcium binding rate constant *k*^+^ and for constant [Ca^2+^]_*eq*_ = 0.1 μM. Each curve corresponds to a fixed total buffer concentration [B]_*T*_ as indicated in the legend. **(B)** Shows that τ increases with increasing equilibrium calcium concentration [Ca^2+^]_*eq*_, and for a fixed total buffer concentration [B]_*T*_ = 10 μM and fixed dissociation constant *K*_*D*_ = 1 μM. Each curve corresponds to a different calcium binding rate constant *k*^+^ (see legend). **(C)** Shows that τ increases with increasing [Ca^2+^]_*eq*_, and for a fixed total buffer concentration [B]_*T*_ = 100 μM and fixed *k*^+^ = 0.1 μM^−1^ ms^−1^. Each curve corresponds to a different buffer dissociation constant *K*_*D*_ (see legend). **(D–F)** In analogy to **(A–C)** these lower row of panels shows the corresponding dependencies of the noise volatility σ. Note that all graphs are shown in log-log coordinates.

The results for the single buffer system are summarized in Figure 1, where the expected noise autocorrelation time τ and noise volatility σ are plotted as functions of different parameters and a simulation volume of *V* = 0.125 fl. Figure 1A shows the dependency of τ on the buffer association rate *k*^+^, at constant [Ca^2+^]_*eq*_ = 0.1 μM. This relationship is computed for different total buffer concentrations [B]_*T*_ (see legend). The shape of the curves can be derived from Equation 22, showing that the autocorrelation time τ decreases with faster buffer kinetics (*k*^+^) and with increasing [B]_*T*_. Figure 1B illustrates the dependency of τ on the equilibrium free calcium concentration [Ca^2+^]_*eq*_ and it is observed that τ is larger at elevated calcium levels. The total buffer concentration was constant at [B]_*T*_ = 10 μM, while the dissociation constant was set to *K*_*D*_ = 1 μM. Each curve represents a different calcium binding rate *k*^+^ (see legend). Another section of the parameter space is explored in Figure 1C, with [Ca^2+^]_*eq*_ as the independent variable again. The total buffer concentration is constant at [B]_*T*_ = 100 μM and the buffer association rate is held constant at *k*^+^ = 0.1 μM^−1^ ms^−1^. This time, each curve corresponds to a different buffer dissociation constant *K*_*D*_ (see legend). The lower set of figures (Figures 1D–F) are analogous in design to Figures 1A–C. Here, the same dependencies are shown with the noise volatility σ as the dependent variable. It is noted that even in the range of physiological buffer kinetics, the noise parameters τ and σ differ substantially.

### 3.2. Numerical results

#### 3.2.1. Single buffer simulations

**Figure 2:**
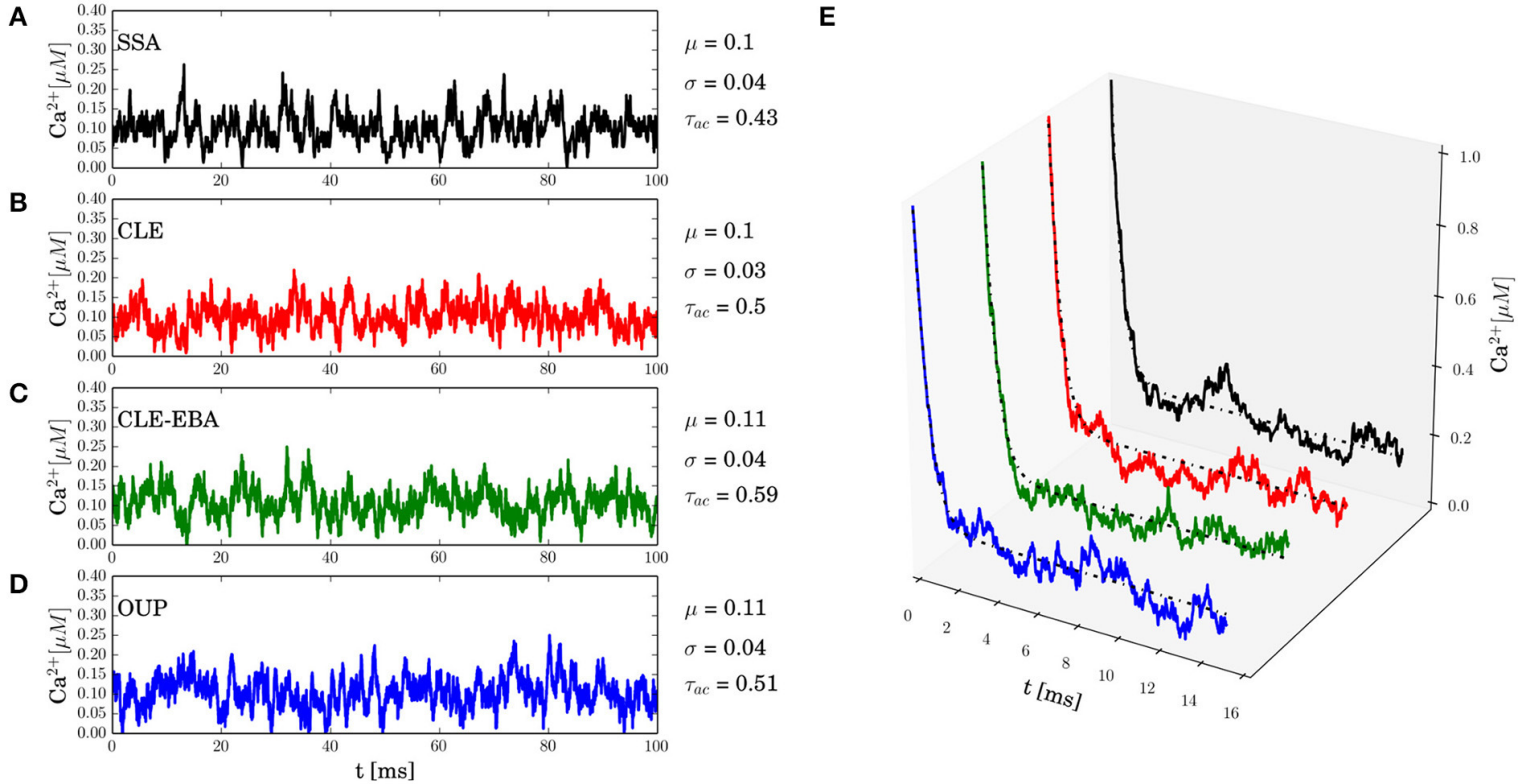
**Equilibrium fluctuations and relaxation from transients**. All simulations represent the calcium-calmodulin single buffer system in a microdomain of *V* = 0.125 fl. The panels on the left side **(A–D)** show equilibrium fluctuations of the free calcium concentration [Ca^2+^] as obtained from different methods. **(A)** Gillespie's exact stochastic simulation algorithm (SSA, black), **(B)** chemical Langevin equation (CLE, red), **(C)** the CLE in the excess buffer approximation (CLE-EBA, green), **(D)** the Ornstein-Uhlenbeck process approximation (OUP, blue). The values of the basic statistical properties analyzed in this paper are given to the right of each panel (mean μ, standard deviation σ, estimated autocorrelation time τ). The data suggests that all methods lead to similar statistical properties. The right panel **(E)** shows the relaxational response of the system to an initial perturbation, [Ca^2+^]_*t* = 0_ = 1 μM and colors indicate the corresponding simulation method (see **A–D**). The analytical solution of the corresponding deterministic system is also shown (dashed black lines). Visually, the decay curves for different simulation methods are nearly identical. A detailed and quantitative analysis of autocorrelation times and decay time constants is given in the main text.

**Figure 3:**
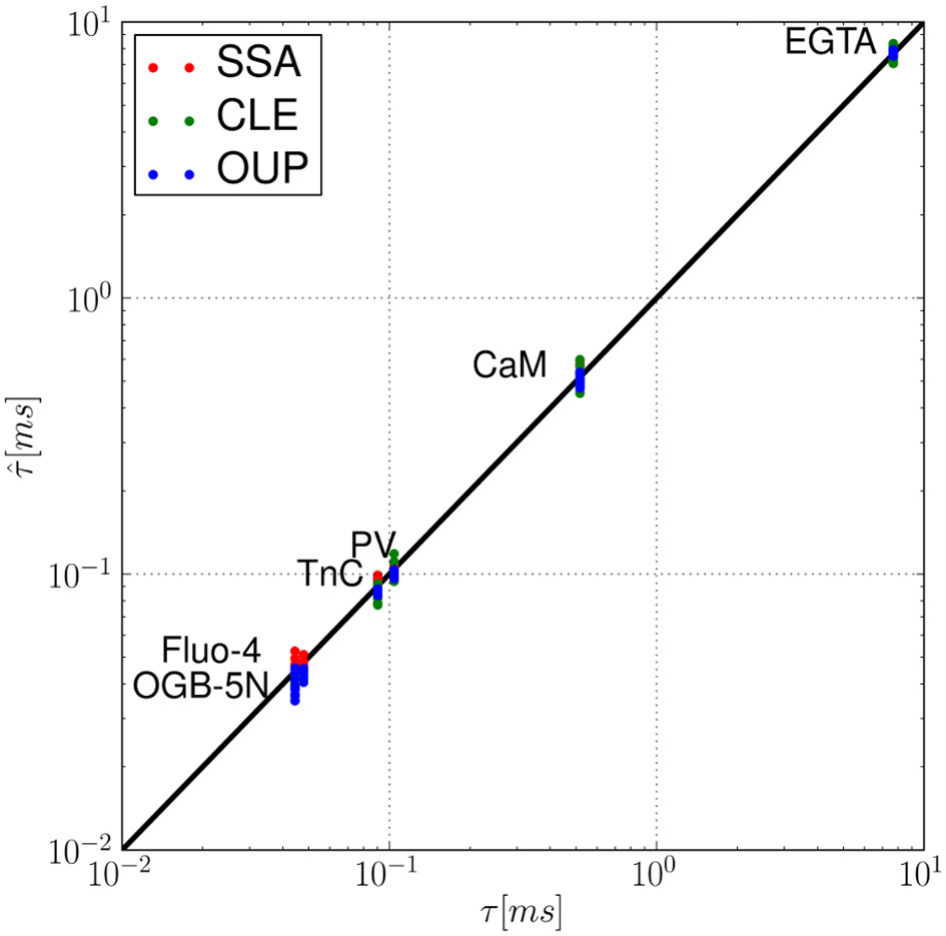
**Fluctuation-dissipation relations in a single buffer system without diffusion: for the set of calcium buffers from Table 1, we compare the noise autocorrelation time τ of equilibrium calcium fluctuations (Equation 22) and the time constant $\hat{\tau }$ that describes the exponential relaxation from a perturbation (Equation 7), e.g., due to the opening of a calcium channel**. For each simulation method (SSA, red; CLE, green; OUP, blue), $\hat{\tau }$ was estimated for *n* = 10 sample paths starting at [Ca^2+^]_*t* = 0_ = 1.0 μM. The proximity to the identity (black line) shows that τ ≈ $\hat{\tau }$.

**Figure 4:**
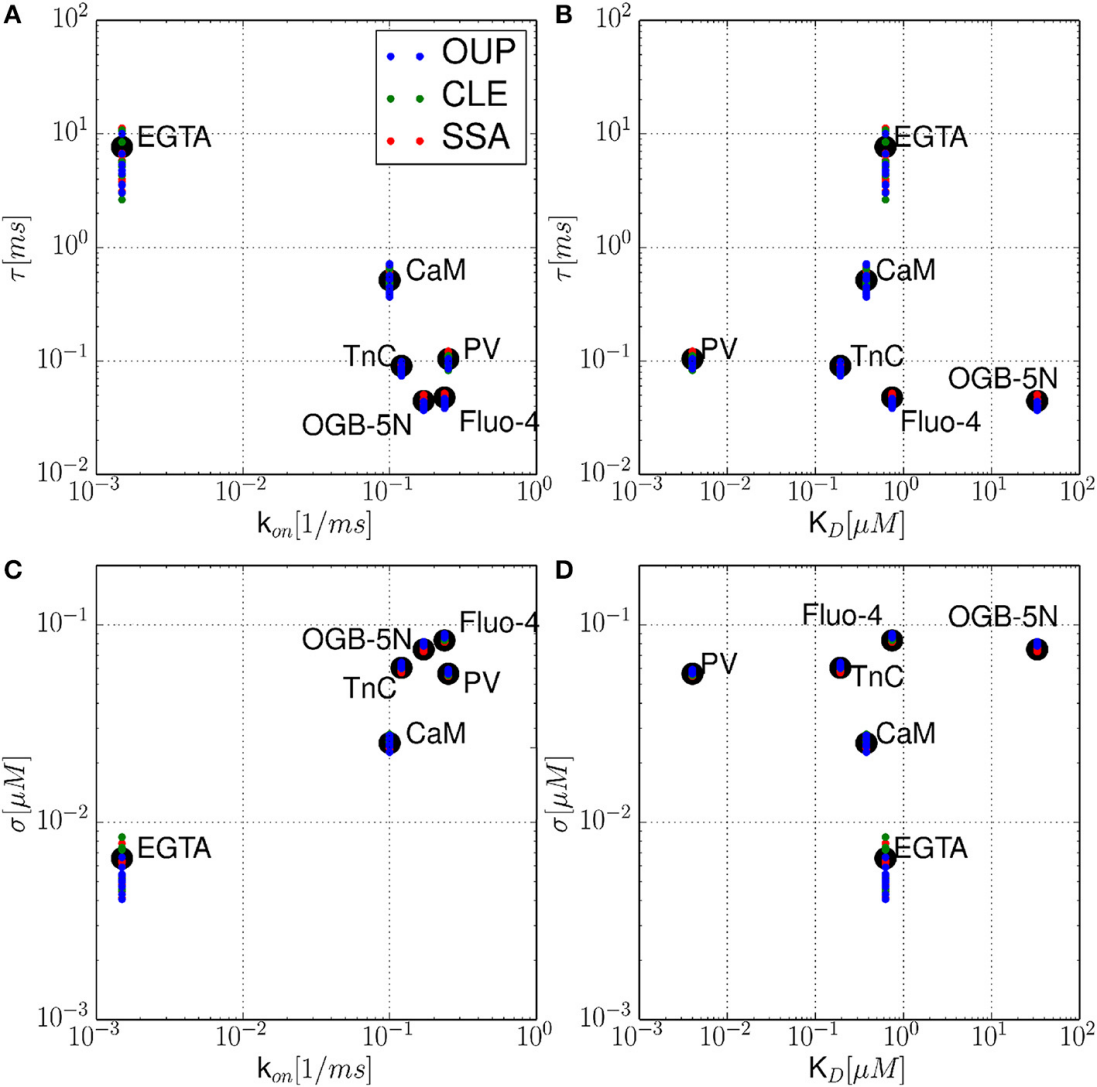
**Noise autocorrelation times τ and volatilities σ for several physiological and artificial buffer species**. The autocorrelation time τ is shown as a function of the calcium binding rate constant *k*^+^ **(A)**, and as a function of the buffer dissociation constant *K*_*D*_ **(B)**, respectively. In the lower row **(C,D)**, the calcium noise volatility σ is shown for the same parameters. It is observed that τ and σ cover a range of several orders of magnitude. Numerical simulations (*n* = 10) using the SSA (red), CLE (green), OUP (blue) are shown as colored dots around the theoretical values (large black dots).

In this section, numerical results for a realistic single buffer system without diffusion are presented. In Figure 2, we chose the calcium-calmodulin system Ca^2+^ + CaM ⇌ Ca^2+^ · CaM in a microdomain of *V* = 0.125 fl and fixed the equilibrium calcium concentration [Ca^2+^] at 0.1 μ*M* for all simulations. On the left side of Figures 2A–D, the intrinsic fluctuations of the local calcium concentration are shown for different simulation methods. From top to bottom, we show the results for Gillespie's exact stochastic simulation algorithm (SSA, black), the chemical Langevin approach (CLE, red), the CLE in the excess buffer approximation (CLE-EBA, green) and the Ornstein-Uhlenbeck approach (OUP, blue). To characterize these sample paths statistically, we show three basic statistical parameters of these processes, i.e., the mean value μ, the process' standard deviation σ and the autocorrelation time τ. Observe that these parameters show a high similarity between the simulation methods tested. Figure 2E shows the relaxational response of the system to a perturbation, where the initial calcium concentration was set to [Ca^2+^]_*t* = 0_ = 1 μ*M*. Again, the response is tested for different methods as indicated by color (see Figures 2A–D). Additionally, the deterministic decay curve as calculated from analytical considerations, Equation 7, is shown (dashed black curves in E). Visual inspection shows a similar shape of the decay curves for the methods tested. Furthermore, it is seen that exact and approximated stochastic results fluctuate around the analytic solution of the deterministic system. In both cases, the results of Gillespie's exact stochastic simulation algorithm (black curves) should be taken as the reference curve for stochastic systems, as the associated algorithm samples the underlying process exactly. At this point, we conclude that the stochastic approximations to Gillespie's exact algorithm do not seem to affect the dynamic and stochastic properties of the single-buffer system significantly. A more detailed and quantitative assessment of this observation is presented in the following paragraphs. The relationship between equilibrium fluctuations and the relaxational response of the system is further analyzed quantitatively in Figure 3. For the frequently occurring calcium buffers given in Table 1, we compare the noise autocorrelation time τ of equilibrium calcium fluctuations, as computed from Equation 22, and the time constant $\hat{\tau }$ that describes the exponential relaxation from an initial perturbation at *t* = 0. The system under consideration has a volume of *V* = 1.0 fl. As seen from Equations 22 and Equation 14, both values should be identical. In a realistic scenario, the perturbation could be due to the opening of a calcium channel. The relaxation time constant $\hat{\tau }$ was estimated from *n* = 10 samples for each simulation method (see legend), and with an initial value of [Ca^2+^]_*t* = 0_ = 1.0 μ*M*. It is observed that the values estimated from all simulations match the expected identity τ = $\hat{\tau }$ (black line) with good accuracy, underscoring the usefulness of the stochastic approximations. Finally, in Figure 4 the expected values of τ and σ for several naturally occurring and artificial buffer species are shown. Note that all values refer to single buffer systems (*V* = 1.0 fl). Figure 4A shows the autocorrelation time τ as a function of the buffer association rate *k*^+^, and as a function of the buffer dissociation constant *K*_*D*_ in B, respectively. The lower row of panels (C, D), σ is shown for the same range of parameters. It is observed that τ and σ cover a range of several orders of magnitude. Numerical simulations (*n* = 10) using the SSA (red), CLE (green), OUP (blue) are shown as colored dots around the theoretical values (large black dots). Simulation results from different algorithms again show a high degree of accuracy and similarity among each other.

#### 3.2.2. Multiple buffers and diffusion

**Figure 5:**
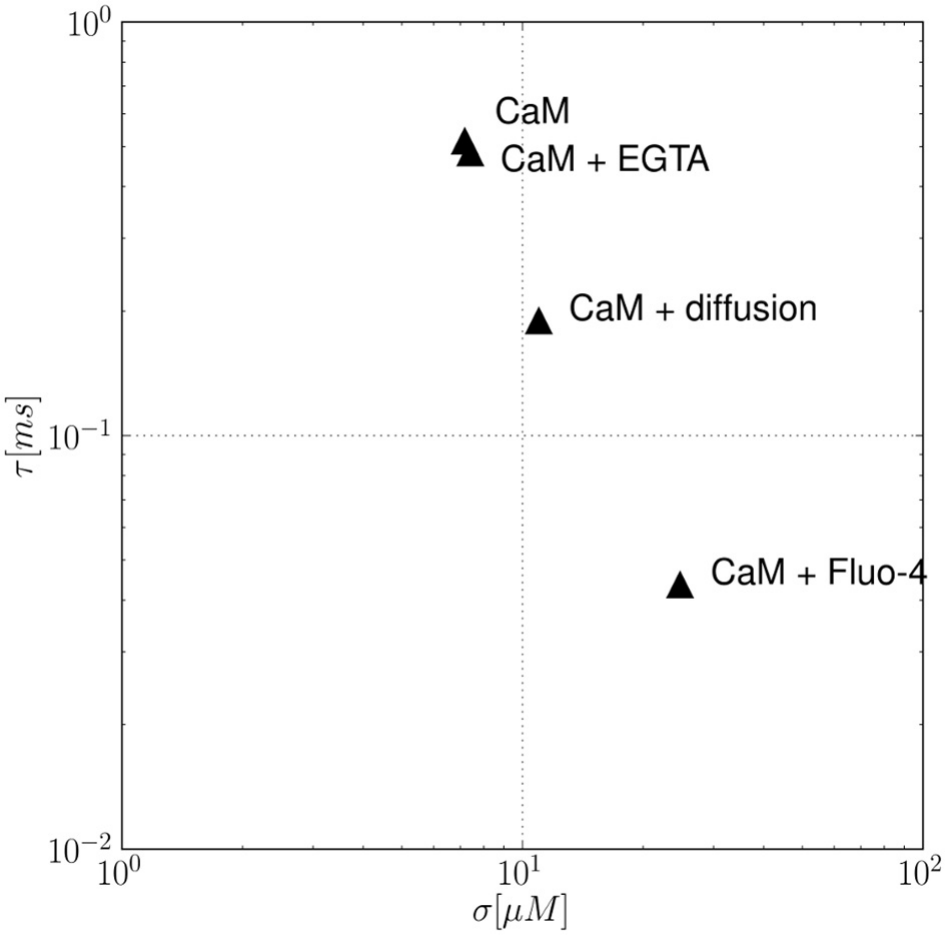
**The effect of multiple buffers and calcium diffusion on the autocorrelation time τ**. In the example, the calcium noise autocorrelation time τ and volatility σ are analyzed for different conditions: a single buffer system (CaM), two double buffer systems (CaM+EGTA, CaM+Fluo-4), and a single buffer (CaM) system with calcium diffusion. Is is observed that the presence of an additional buffer leads to a faster decorrelation of the fluctuating calcium concentration, i.e., smaller τ values. Analogously, including diffusion effects also decreases τ values.

In order to add complexity and simulate a more realistic situation, the effect of multiple buffers and calcium diffusion on the autocorrelation time τ is analyzed in Figure 5. In the given example, τ and σ are analyzed for (i) a single buffer system (CaM), (ii) two different systems containing two buffers each (CaM+EGTA, CaM+Fluo-4), and (iii) a single buffer(CaM) system with calcium diffusion. Comparing the kinetic constants of the different buffers as given in Table 1, we see that both, EGTA and Fluo-4, share a similar calcium affinity. However, the association and dissociation events to and from Fluo-4 occur much faster than for EGTA. The constants as found in the literature differ by an approximate factor of 100. Consequently, the autocorrelation time of the CaM+EGTA system is mainly determined by the fast CaM dynamics and is hardly changed by addition of the two slow EGTA reactions. The comparatively few binding and dissociation events between Ca^2+^ and EGTA that can occur in a given time interval accelerate the decorrelation of the calcium fluctuations only slightly. Therefore, the autocorrelation time of the combined buffer system is smaller, but almost identical to the single buffer system containing CaM only (τ_*CaM*_ = 0.516 ms, τ_*CaM,EGTA*_ = 0.483 ms). In the case of adding Fluo-4 however, we find major changes in the statistical properties of the resulting calcium signal. Table 1 shows that especially the dissociation constant of the Ca-Fluo-4 complex is significantly larger than that of the Ca-CaM complex. The faster pace of reactions leads to an earlier decorrelation of the signal as shown by the resulting autocorrelation times (τ_*CaM,Fluo*−4_ = 0.044 ms). The magnitude of diffusion effects lies in-between the effects of EGTA and Fluo-4, in the present example. The reader is warned not to generalize the relative effect of buffers and diffusion too quickly. From our derivation, it is explicitly clear that the effects also depend on the absolute concentration of buffers and calcium, respectively. In our case, the calcium diffusion constant of 200 μ*m*^2^/*s* at a mean calcium concentration of 100 nM leads to an autocorrelation time of τ_*CaM,Diff*._ = 0.190. At the same time, the noise volatility σ is influenced in the opposite direction. Clearly, the number of possible parameter combinations grows rapidly with the number of parameters allowed. The current article does not aim to scan this parameter space exhaustively.

#### 3.2.3. A colored noise-induced transition

In order to illustrate a potential physiological role of autocorrelated calcium noise, we revisit a generic example for the study of colored noise effects on dynamical systems. In particular, we consider the one-dimensional dynamics of a single variable *X*_*t*_ moving in a bistable potential represented by a fourth-order polynomial $V(X)=-\frac{a}{2}X^{2}+\frac{b}{4}X^{4}$ (Haenggi and Jung, 1995). The dynamics is driven by a colored noise process ξ_*t*_ representing calcium noise, and integration is performed using a first-order Euler scheme with *dt* = 0.01 ms. The equation of motion is given by

(24) $$dX_{t}=-\partial_{X}V(X_{t})\;dt+\xi_{t}.$$

**Figure 6:**
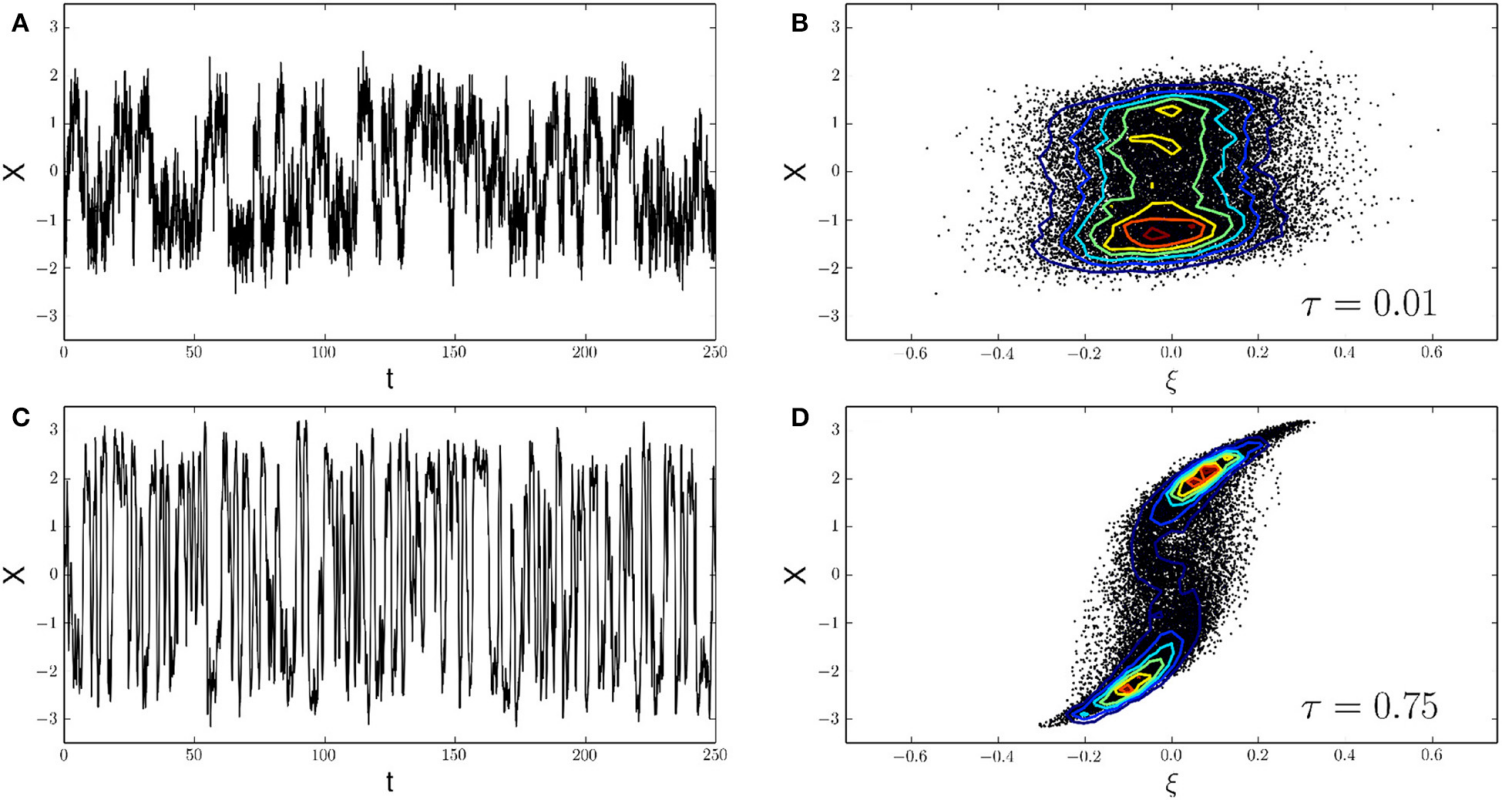
**A colored noise-induced transition**. **(A,C)** The continuous gating of a calcium-sensitive ion channel can be reduced to the motion of a single particle *X*_*t*_ in a double-well potential. *X*_*t*_ is the channel variable and ξ_*t*_ is the calcium noise variable driving the motion of *X*_*t*_ (see Equation 24). The shape of the potential then leads to characteristic time series with “on-off” kinetics as observed in **(A)** (τ = 0.01) and **(C)** (τ = 0.75). Here, we assume the closed-open transition of the channel to be calcium-dependent. Calcium noise ξ_*t*_ is implemented with the OUP method (other methods yield qualitatively identical results, not shown). **(B,D)** Correlations between the calcium noise ξ and the channel state variable *X* are analyzed via their empirical joint probability distribution *P*(ξ, *X*). For a short autocorrelation time of the noise, τ = 0.01, the joint distribution is almost symmetrical around the origin, indicating that the noise and the channel variables have a low correlation **(B)**. At τ = 0.75 however, the joint distribution is clearly skewed. This indicates a strong positive correlation between both variables **(D)**. Such correlations between microdomain calcium noise and the functional state of calcium-sensitive target molecules, controlled by the autocorrelation time of calcium noise, await experimental validation.

The system can represent the generalized dynamics of an ion channel whose two functional states (open, closed) are represented by the local minima of the potential (Liebovitch and Czegledy, 1992). The system is an abstract representation of bistable dynamics and can also represent more involved biochemical systems whose common feature is bistability. Figure 6 shows the results of two simulation runs, both using the Ornstein-Uhlenbeck approximation of calcium noise as the “noisy drive” ξ_*t*_ of the target variable *X*_*t*_. To assess the effects of noise autocorrelation time, we used two different driving signals, one with a small autocorrelation time of τ = 0.01 (Figures 6A,C), and another one using τ = 0.75 (Figures 6B,D). By comparison with our previous results, we see that both τ-values fall within the range of autocorrelation times obtained with natural calcium buffers. In both cases, the noisy drive ξ_*t*_ induces a motion of the variable *X*_*t*_ that shows bistable (or “on-off”) kinetics due to the double-well shape of the potential. Using the analogy of a calcium-regulated ion channel, the “on-off” kinetics of the variable *X*_*t*_ represents the continuous gating of the calcium sensitive ion channel and we assume that the closed-open transition is calcium-dependent. To show the influence of noise correlations on channel gating, we look at the empirical joint probability distribution *P*(ξ, *X*) of the noise variable ξ_*t*_ and the channel variable *X*_*t*_. At τ = 0.01, the joint distribution is almost radially symmetric around the origin, indicating a lack of correlation between the noise ξ_*t*_ and the channel variable *X*_*t*_. In other words, the joint distribution approximately factorizes as *P*(ξ, *X*) ~ *P*(ξ) × *P*(*X*). At τ = 0.75 however, the result is a heavily skewed joint distribution, showing a strong coupling of channel gating (*X*_*t*_) to the correlated calcium noise (ξ_*t*_). It could be argued that this effect only occurs for the Ornstein-Uhlenbeck approximation of calcium noise. We also tested other algorithms (Gillespie's algorithm, chemical Langevin equation) to simulate ξ_*t*_ and observed the same qualitative effects (data not shown). Please note that in the present example, the mean value of the noise was removed to comply with the symmetry of *V* around the origin. Furthermore, in the example shown only τ was varied, keeping σ constant. This leads to differing variances for the noise processes, however, when correcting for this, the described effects persist.

## 4. Discussion

In the present article, we analyze stochastic simulation strategies for intracellular calcium microdomains. In a previous article, we investigated the potential of Gillespie's exact stochastic simulation algorithm to simulate calcium microdomains containing buffers and calcium-regulated calcium channels (von Wegner and Fink, 2010). Here, we focus on the properties of calcium noise itself. Already in our previous work, we assessed the effects of including diffusion events in the simulation by analyzing the autocorrelation time and the power spectral density of calcium noise. However, while our previous investigations were focused on accuracy, therefore implementing Gillespie's exact algorithm, we here concentrate on approximations to the exact solution of the chemical master equation. In particular, we use the chemical Langevin equation (Gillespie, 2000) and apply the excess buffer approximation (Heinemann et al., 1986; Smith et al., 2001; Fall et al., 2002) to simplify our results. We find that close to the resting calcium concentration, the excess buffer approximation (EBA) is a valid assumption over the range of physiological buffer concentrations. Fluctuations around the equilibrium buffer concentrations are very small, yielding coefficients of variation smaller than 10^−3^ (Table 1). For more complex situations, including Ca^2+^ channels for instance, it has to be remembered that buffer saturation can occur close to the channel pore, thereby invalidating the EBA assumption (Smith, 1996). We are aware of only one very recent publication using the chemical Langevin equation to analyze stochastic fluctuations of the calcium concentration in a microdomain and the influence of calcium buffers (Weinberg and Smith, 2014). For whole cell models, the chemical Langevin equation has been used more often (Li et al., 2005; Manninen et al., 2006). Other stochastic approaches for microdomains are based on explicit molecular random walk simulations (Franks et al., 2002; Shahrezaei and Delaney, 2004; Keller et al., 2008) and on the Fokker-Planck representation of the chemical master equation. The latter has been used to simulate calcium microdomains as found in the dyadic cleft of cardiac myocytes (Winslow et al., 2006; Hake and Lines, 2008).

The main focus of the present work are the autocorrelation properties of calcium noise, i.e., the noise “color.” Examples from many scientific areas show that noise color alone can determine a systems behavior and can even eventuate transitions between distinct stable states (Haenggi and Jung, 1995). However, so far there is no experimental evidence showing a functional role of calcium noise properties within a microdomain reaction network. To obtain quantitative results, we formulate the chemical Langevin equations of simplified model systems containing calcium ions, buffers and diffusion events. Combining noise terms and using the excess buffer approximation, we obtain surrogate processes that can be characterized by few parameters (μ, τ, σ), similar to an Ornstein-Uhlenbeck process. In some expressions, non-stationary terms were further substituted by their expected values at chemical equilibrium and thus, stationary expressions were obtained. With these expressions, the influence of buffer kinetic parameters and the calcium diffusion constant were quantified and illustrated for a section of the physiologically plausible parameter space. To summarize, we are able to characterize microdomain calcium noise as a colored noise process, noise color being characterized by physico-chemical system properties. In terms of computational speed, the chemical Langevin equation and the approximations presented here represent a major improvement in performance. Using the Ornstein-Uhlenbeck type approximations, the implementation of calcium noise is elementary and integration of realistic calcium noise into other simulation frameworks reduces to adding one further equation. In the context of systems described by ordinary or stochastic differential equations, adding calcium noise does not change the simulation framework conceptually as would be necessary when using Gillespie's algorithm.

To discuss situations in which the statistical properties of local calcium noise may be of physiological relevance, we chose a well studied example of a noise-induced transition in a generic bistable system (Figure 6, Haenggi and Jung, 1995). Bistability is a dynamical feature frequently encountered in biological systems, especially in intracellular signaling networks. Prominent examples are given by the genetic toggle switch (Tian and Burrage, 2006), phosphorylation-dephosphorylation networks (Kholodenko, 2006) and abstract representations of ion channels (Liebovitch and Czegledy, 1992). Each simulation method to generate calcium noise, namely Gillespie's algorithm, the chemical Langevin equation, and the Ornstein-Uhlenbeck representation, yield results analogous to Figure 6. The simulations show a coupling of the bistable variable to the noise process, or in terms of a more biological interpretation, a possible coupling of a calcium-regulated ion channel to the local calcium noise surrounding the channel. Research on more specific examples including realistic calcium channels and complex buffer situations are currently under way. Previously published complex models of cellular signaling networks driven by calcium implemented calcium noise as a random flux with a fixed rate (Bhalla, 2004). As our results suggest a possible functional role of calcium noise properties on calcium-sensitive reaction networks, the calcium noise implementation should be chosen carefully. Our results show how system parameters can be used to calculate realistic noise parameters.

In contrast to a full stochastic description of the system (Gillespie's algorithm), the Langevin method approximates the number of reactions in a fixed time interval as an appropiately scaled, normally distributed random variable with identical mean and variance. The principal limitation of the method becomes apparent with small reaction volumes, where the choice of a normally distributed variable can lead to physically meaningless negative molecule counts. While the solution to this problem would be to use the stochastically and physically correct Poisson distribution, the Langevin approximation has deeper theoretical connections and provides formulae for spectral properties and the cross-correlation structure of multi-variable systems (Gillespie, 2000; Gardiner, 2009; Weinberg and Smith, 2014). However, the validity of the Langevin approach has to be validated for each new model. A yet unexplored, but possibly interesting alternative could arise from the stochastic Cox-Ingersoll-Ross process (Goeing-Jaeschke and Yor, 2003). The process is also mean-reverting and its volatility σ_*t*_ scales as $\sqrt{X_{t}}$, similar to Equation 15.

Finally, the numerical predictions presented should receive further attention by experimentalists. An interesting perspective in this context is the possibility of performing measurements of localized calcium fluctuations by means of a fluorescent calcium indicator dye and a stationary laser, namely a fluorescent correlation spectroscopy (FCS) setup. To the best of our knowledge, Ca^2+^-FCS measurements have not been performed yet but the numerical results indicate that such a setup could be feasible. Such measurements would allow to quantify the magnitude and correlation structure of calcium flucutations in different cellular compartments and would allow conclusions about the buffer composition. In summary, as the attention of researchers shifts toward the stochastic nature of calcium dynamics, novel insights on the regulation of microdomain signaling networks are expected.

### Conflict of interest statement

The authors declare that the research was conducted in the absence of any commercial or financial relationships that could be construed as a potential conflict of interest.
